# Lifetime exposure to bullying and its association with health status and quality of life in the general Norwegian population: a cross-sectional study

**DOI:** 10.3389/fpubh.2026.1811310

**Published:** 2026-04-20

**Authors:** Laila Skogstad, Inger Schou-Bredal, Øivind Ekeberg, Tine K. Grimholt, Tore Bonsaksen, Trond Heir

**Affiliations:** 1 Centre for Clinical Heart and Lung Research, Department of Thoracic Surgery, Division of Cardiovascular and Pulmonary Diseases, Oslo University Hospital, Oslo, Norway; 2 Faculty of Medicine, Institute of Health and Society, University of Oslo, Oslo, Norway; 3 Private Practitioner, Oslo, Norway; 4 Department of Health, VID Specialized University, Oslo, Norway; 5 Department of Health and Nursing Sciences, Faculty of Social and Health Sciences, University of Inland Norway, Elverum, Norway; 6 Department of Health, VID Specialized University, Stavanger, Norway; 7 Institute of Clinical Medicine, University of Oslo, Oslo, Norway

**Keywords:** bullying, epidemiology, general population survey, physical health, psychological health, quality of life

## Abstract

**Background:**

Bullying may have a considerable negative impact on health and well-being. Nevertheless, there is a lack of studies investigating exposure to bullying in the general population. Thus, we aimed to assess the prevalence of lifetime exposure to bullying in the adult Norwegian population and to compare health-related quality of life (HRQoL), and physical and psychological health problems between bullied and non-bullied.

**Methods:**

We measured lifetime exposure to bullying in the general Norwegian population using a self-report questionnaire. The study was part of a cross-sectional national health survey (*n* = 1733) conducted in 2014–2015, to which a probability sample of people in the general population was recruited. Reports on socio-demographic data, HRQoL and a wide range of mental and somatic health problems provided the basis for a comparison of those who were bullied with those who were not. Chi square tests and logistic regression analyses were used to assess associations with lifetime exposure to bullying.

**Results:**

Across age groups, 28.2% reported exposure to bullying during their lifespan. Adjusted for other variables, the odds of bullying were higher in younger age groups, for those who lived as singles, and for those who were not working or in education. Respondents who had been bullied reported more mental problems such as depression, anxiety, insomnia, eating disorder, psychosis, self-harm, and suicide attempts, and they had lower HRQoL. They also reported more chronic pain, fibromyalgia, rheumatoid arthritis, diabetes mellitus, obesity, and musculoskeletal-, respiratory- and gastrointestinal diseases than people who had not been exposed to bullying.

**Conclusion:**

Bullying is common in the general Norwegian population, where more than a quarter of respondents had experienced exposure to bullying during their lifespan. Substantial more mental and somatic health problems among those exposed to bullying require a broad effort against bullying in central arenas in society.

## Background

Bullying is an extreme form of systematic and long-lasting social degradation or alienation that exceeds the limits of normative behavior (1). Olweus (2) has defined exposure to bullying as perceiving negative actions from one or more people, repeatedly and over time, and where the target of the bullying finds it difficult to defend themselves. Bullying includes several types of abuse, both psychological, verbal, and physical (3).

Research on bullying has largely been restricted to either populations of school-age children or to adults in working life. A large cross-national study showed that the proportion of 11-15-year-old children who reported bullying in the last 2 months was 29%, varying from 8 to 60% across the European countries (4). The prevalence of bullying in working life has been documented in a worldwide meta-analysis in which 11 to 18% of employees perceived themselves as victims of bullying (5).

Studies on bullying through all phases of life are rare. Such studies are important because bullying is not limited to children of school age or adults in working life but occurs in all social arenas and in all age groups. A population-based study in South Australia has revealed that 45.6% of the Australian population had been exposed to bullying during their lifetime (6). Corresponding prevalence in a nationwide population-based study in Taiwan was 13.5% (7). Substantial between-country differences make it necessary to examine the prevalence of bullying in more countries and cultures.

Bullying may have a considerable negative impact on the health and well-being of those being affected. In large studies, exposure to bullying in formative years has been found to be related to poorer health, both concurrently (8) and longitudinally (9). Studies of bullying in school or working life have consistently shown a higher risk of mental health problems, including depression (10, 11), insomnia (12, 13), anxiety (9, 14), eating disorder (15), self-harm (16), and suicidal ideation (17, 18) among those who have been exposed to bullying. Similar studies have also shown a higher risk of somatic health problems such as chronic pain (19, 20), fibromyalgia (21), diabetes mellitus (22), asthma (23), and physician-diagnosed respiratory disease (24).

Due to a limited number of studies, the same health variables have been insufficiently investigated in population-based surveys consisting of people of all ages and bullying in a lifelong perspective. In a population-based survey in Taiwan, exposure to bullying was associated with general psychopathology, lifetime suicide ideation and suicide attempt (7). In a similar survey from South Australia, bullying exposure was associated with reduced health-related quality of life, excessive alcohol intake, binge eating, and antidepressant use (6).

The purpose of the study was twofold; firstly, to investigate the lifetime prevalence of being exposed to bullying in the adult Norwegian population. Secondly, we wanted to compare the mental and somatic health status between those who had been exposed to bullying and those who had not.

## Methods

### Design

This study of exposure to bullying was part of the Norwegian Population Health Survey, which was established to obtain knowledge about a wide range of health-related characteristics of the population (25, 26). The data collection took place in 2014–2015. The survey used a cross-sectional survey design based on a national probability sample constructed by the Central National Register of Norway. Names and addresses of 5,500 individuals were randomly selected from a public registry of the Norwegian adult population, stratified by age, gender and region of residence, aiming to be representative of the general population.

### Participants

**Figure 1:**
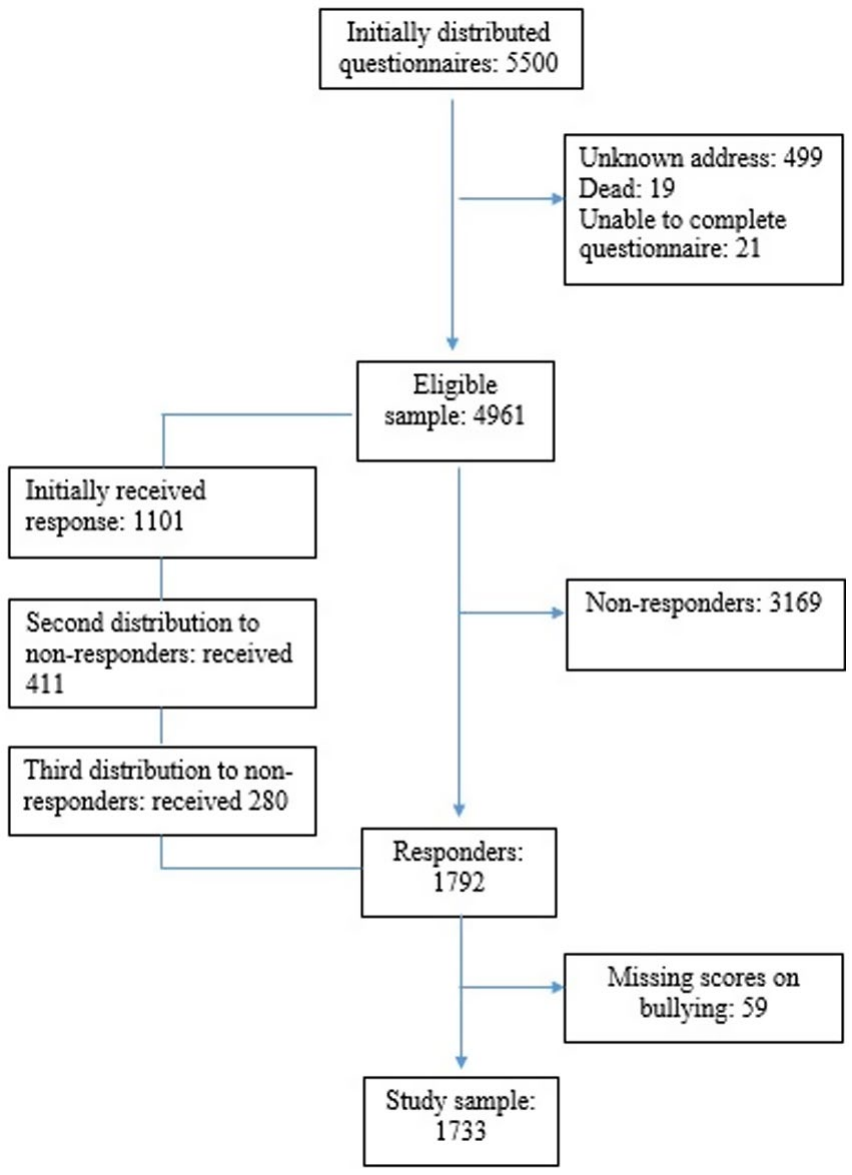
Flowchart showing the inclusion of the participants.

Eligible participants were Norwegian citizens, 18 years or older, and able to read and understand the language used in the survey (Norwegian). A questionnaire along with a letter detailing the purpose of the study, survey procedures and participants’ rights were sent by post to 5,500 randomly selected individuals stratified by age, gender and geographic region, for possible inclusion in the study. Feedback about respondents who had died (*n* = 9), were unable to complete the questionnaire due to illness or old age (*n* = 21) or could not be reached due to an invalid address (*n* = 499) left an eligible sample of maximum 4,961 available individuals (Figure 1).

We made up to three attempts to contact non-responders. In total, 1792 persons accepted to take part in the study and completed the questionnaire. Of these, 59 had missing responses to the question about bullying, rendering a final sample of 1733 participants included in the analyses. The survey was carried out anonymously. Upon request, the Regional Committees for Medical Research Ethics Southeast Norway had no objections to the study (REK 2014/719). The principles in the Declaration of Helsinki were respected.

### Measures

We obtained general data about age, gender, municipality of residence, marital status, educational level, and occupational status. For the present study we collected data on lifetime exposure to bullying. As part of the larger health survey, we also collected data on the respondent’s health status and quality of life.

Lifetime exposure to bullying was assessed by a single-item question retrieved from the General Nordic Questionnaire for Psychological and Social Factors at Work (27): ‘Have you ever been exposed to bullying (in school, at your workplace or any other place)?’ Bullying was defined as offensive behavior that had to occur repeatedly over a period, and the person confronted had to experience difficulties defending himself/herself. The response categories were no or yes. This single-item approach is considered a valid measurement of bullying (28).

The respondents were presented with a list of mental and somatic health problems and answered yes or no to whether they currently or previously had each of the conditions presented. The list included seven mental disorders (depression, anxiety, insomnia, eating disorder, psychosis, history of self-harm or suicide attempts) and 13 somatic diseases (chronic pain, gastrointestinal disease, prolonged musculoskeletal disorder, respiratory disease, fibromyalgia, diabetes mellitus, rheumatoid arthritis, tinnitus, arthrosis, heart disease, cancer, osteoporosis, stroke). We also defined which participants suffered from obesity (BMI > 30), based on what they reported when asked about height and weight. BMI was calculated as the weight (kg) divided by the square of the height (m^2^).

Health-related quality of life (HRQoL) was assessed with the following two items from the European Organization for Research and Treatment of Cancer questionnaire (29): ‘How has your health been during the last week?’ and (ii) ‘How has your quality of life been during the last week?’ For both questions we used an 11–point scale ranging from “extremely poor” (0) to “excellent” (10). HRQoL was given as the average of the two items multiplied by 10.

### Statistical analysis

We presented proportions belonging to various socio-demographic groups as well as proportions with different health problems as percentages (with exact numbers in parentheses) for bullied and non-bullied individuals separately. Statistical comparisons were based on chi-square tests. We used multiple logistic regression with socio-demographic variables as independent and bullying as dependent variable to estimate the distribution of perceived bullying in the population.

We also used multiple logistic regression to adjust for age, gender, marital status, education and occupational status in the association between perceived bullying (independent variable) and each of the health problems (dependent variable). We limited the last analysis to those health problems that had significant differences between bullied and non-bullied individuals in the unadjusted comparison.

Group differences in levels of health-related quality of life were estimated by the independent *t*-test. All tests were two-tailed, and differences were considered significant if *p* < 0.05. Data were analysed using SPSS for Windows version 24 (SPSS Inc., Chicago, IL, USA).

## Results

Respondents who answered the question about bullying made up 34.9% of the eligible sample (Figure 1). Both for those who reported bullying and those who did not, there was a relative deficit of respondents in the youngest age groups (18–30 and 31–40 years) and among men (Table 1).

**Table 1:** Sociodemographic data for bullied and non-bullied respondents.

Variables	Bullied (*n* = 489)	Not bullied (*n* = 1,244)	*p*-Value
Age group	% (*n*)	% (*n*)	<0.001
18–30	16.7 (81)	9.9 (122)	
31–40	14.0 (68)	9.1 (112)	
41–50	21.6 (105)	19.6 (242)	
51–60	22.7 (110)	19.1 (235)	
61–70	17.3 (84)	24.5 (302)	
71+	7.6 (37)	17.8 (219)	
Gender			0.49
Male	46.7 (227)	46.8 (579)	
Female	53.3 (260)	53.2 (658)	
Municipality of residence			0.37
Village with <2000 inhabitants	20.9 (101)	20.2 (248)	
Village with ≥2000–19.999	26.2 (127)	28.1 (345)	
City with 20.000–99.000	23.1 (112)	24.6 (302)	
City with ≥100.000 or more	29.8 (144)	27.1 (332)	
Marital status			<0.001
Married or cohabitated	64.6 (316)	74.4 (925)	
Single	35.4 (173)	25.6 (319)	
Educational level			0.098
Higher education level (>12 years)	56.7 (276)	53.1 (655)	
Lower education level (≤12 years)	43.3 (211)	46.9 (579)	
Occupational status			0.003
Working or undergoing education	70.6 (345)	63.3 (788)	
Not working	29.4 (144)	36.7 (456)	

0–6% missing within the different sociodemographic variables.

### Prevalence of bullying

The lifetime prevalence of being exposed to bullying was 28.2%. Table 2 shows associations between sociodemographic variables and exposure to bullying. Perceived bullying was most common in the two youngest age groups, 18–30 years (39.9%) and 31–40 years (37.8%), and least common in the two oldest age groups, 61–70 years (21.8%) and those over 70 years (14.5%). There were insignificant differences in prevalence between women and men, nor were there any differences regarding municipality of residence.

**Table 2:** Logistic regression analyses showing associations between sociodemographic variables (independent) and exposure to bullying (dependent variable).

Variables	Univariate			Multivariate		
	OR	95% CI	*p*-Value	OR	95% CI	*p*-Value
Age group						
18–30 years	Reference			Reference		
31–40 years	0.91	0.61–1.38	0.67	1.07	0.69–1.66	0.77
41–50 years	0.65	0.46–0.94	0.02	0.77	0.52–1.14	0.19
51–60 years	0.71	0.49–1.01	0.06	0.80	0.54–1.18	0.25
61–70 years	0.42	0.29–0.61	<0.001	0.37	0.24–0.58	<0.001
71+ years	0.25	0.16–0.40	<0.001	0.19	0.11–0.32	<0.001
Gender						
Male	Reference			Reference		
Female	1.01	0.82–1.24	0.94	0.88	0.71–1.10	0.27
Size of place of living						
<2,000 inhabitants	Reference			Reference		
2,000–19,999 inhabitants	0.90	0.66–1.22	0.48	0.76	0.55–1.04	0.09
20,000–99,999 inhabitants	0.90	0.66–1.24	0.52	0.81	0.58–1.13	0.21
≥100,000 inhabitants	1.06	0.78–1.43	0.73	0.81	0.58–1.12	0.19
Marital status						
Spouse/partner	Reference			Reference		
Not spouse/partner	1.59	1.27–1.99	<0.001	1.61	1.25–2.07	<0.001
Education level						
High school or lower	Reference			Reference		
Higher education	1.16	0.94–1.43	0.18	1.11	0.88–1.41	0.36
Occupational status						
Working or in education	Reference			Reference		
Not working/in education	0.72	0.58–0.90	0.005	1.63	1.18–2.24	0.003

Those who lived as singles had experienced more bullying (35.2%) than those who were married or cohabiting (25.5%). There were no significant differences when it came to level of education completed, while the experience of being bullied was more common among those who were currently in work or in education (30.5%) than among those who were not (24.0%). When adjusting for other variables, multivariate logistic regression analysis confirmed the results from the unadjusted analyses, except for occupational status. In contrast to the unadjusted analysis, the odds of lifetime exposure of bullying were higher for those who were not working or in education (Table 2).

### Health problems and life quality

Table 3 shows mental and somatic health problems among bullied and non-bullied individuals. Respondents who reported bullying had more mental health problems of all categories. They also had more psychosomatic and somatic complaints such as chronic pain, gastrointestinal disease, obesity, musculoskeletal disorder, respiratory disease, fibromyalgia, diabetes mellitus and rheumatoid arthritis.

**Table 3:** Proportions with various health problems among the bullied and non-bullied.

	Bullied (*n* = 489)	Not bullied (*n* = 1,244)	*p*
	% (*n*)	% (*n*)	
Mental health problems			
Depression	45.1 (216)	23.8 (289)	<0.001
Anxiety	31.5 (151)	18.0 (217)	<0.001
Insomnia	48.9 (234)	33.4 (407)	<0.001
Eating disorder	15.4 (73)	4.9 (59)	<0.001
Psychosis	3.4 (16)	1.4 (16)	0.017
History of self-harm	10.2 (49)	1.2 (15)	<0.001
History of suicide attempts	6.6 (32)	1.6 (19)	<0.001
Somatic health problems			
Chronic pain	31.7 (147)	19.8 (234)	<0.001
Gastrointestinal disease	22.5 (102)	16.5 (192)	0.004
Obesity (BMI ≥ 30)	20.6 (94)	14.2 (167)	0.007
Musculoskeletal disorder	17.8 (81)	12.5 (146)	0.001
Respiratory disease	16.4 (75)	11.0 (129)	0.013
Fibromyalgia	7.7 (35)	3.5 (41)	0.001
Diabetes mellitus	7.3 (33)	5.0 (59)	0.002
Rheumatoid arthritis	6.9 (31)	4.7 (54)	0.033
Tinnitus	21.8 (100)	17.8 (209)	0.101
Arthrosis	19.0 (88)	17.3 (203)	0.215
Heart disease	9.2 (42)	11.9 (140)	0.263
Cancer	8.2 (37)	8.4 (99)	0.548
Osteoporosis	3.1 (14)	3.6 (41)	0.880
Stroke	2.2 (10)	3.0 (35)	0.552

Percentages are provided as valid percent. Missing data ranging between 1.7% (suicide attempts) and 6.9% (rheumatoid arthritis).

All differences in health problems between bullied and non-bullied remained significant after adjustment for age, gender, marital status, educational level, and occupational status (Table 4). As Table 4 shows, the association between bullying and health problems was most prominent for self-harm and suicide attempts, but also conspicuous for several other conditions.

**Table 4:** Logistic regression analyses for associations between exposure to bullying (independent variable) and mental or somatic health problems (dependent variables) when adjusted for age, gender, marital status, education level and employment status.

	OR	CI 95%	*p*
Mental health problems			
Depression	2.45	1.94–3.09	<0.001
Anxiety	1.94	1.51–2.50	<0.001
Insomnia	1.91	1.53–2.34	<0.001
Eating disorder	2.92	1.99–4.29	<0.001
Psychosis	2.30	1.10–4.81	0.027
History of self-harm	6.82	3.68–12.62	<0.001
History of suicide attempts	4.18	2.27–7.69	<0.001
Somatic health problems			
Chronic pain	2.14	1.66–2.76	<0.001
Gastrointestinal disease	1.60	1.21–2.12	0.001
Obesity (BMI > 30)	1.63	1.22–2.17	0.001
Musculoskeletal disorder	1.77	1.29–2.41	<0.001
Respiratory disease	1.59	1.16–2.19	0.004
Fibromyalgia	2.72	1.61–4.59	<0.001
Diabetes mellitus	1.70	1.04–2.79	0.035
Rheumatoid arthritis	1.90	1.39–2.59	<0.001

Health-related quality of life was generally lower in those who had been exposed to bullying compared to those who had not (70.7 versus 77.6, *p* < 0.001).

## Discussion

In this cross-sectional study of the general Norwegian population, more than a quarter of the respondents (28.2%) reported that they had been exposed to bullying during their lifespan. Bullying was more often reported in younger age groups, by people who lived without a spouse or partner, and by those who were neither in work nor in education. People who reported lifetime exposure to bullying had more psychological problems and more psychosomatic and somatic complaints, including a wide range of psychiatric and somatic disorders. Self-reported quality of life was also lower for people who had been exposed to bullying during their lifetime.

Compared to a limited number of other population studies that have examined lifetime exposure to bullying, our estimated 28.2% prevalence was higher than in Taiwan (13.5%) (7) and lower than in Australia (45.6%) (6). Substantial differences between countries can, in addition to actual variations in negative behavior, be due to cultural differences in what people experience as bullying or by diverging definitions of bullying in the studies.

The association between lifetime bullying and the respondents’ age, with a decline in prevalence with increasing age, may be compared to findings in an Australian study of previous exposure to school bullying (30). In that study, younger generations were more likely to report that they had been bullied at school than the older ones. Even though our study also included bullying in adulthood and the possibility of exposure is assumed to increase with years of life, the difference between the age groups was at least as convincing in our study. The frequency of bullying, especially in younger age groups, may of course have increased over time, or older age groups may have forgotten that they were exposed to bullying. But it may also be that younger people today label more negative behavior as bullying or that they find it easier to define themselves as bullied (30).

The associations shown between lifetime exposure to bullying and marital or occupational status are consistent with previous findings that people who have experienced bullying in childhood have lower social functioning in adulthood, lower educational attainment, lower employment rates and a weaker connection to the labor market (31, 32). The findings can be interpreted in the light of knowledge that being exposed to bullying can affect self-esteem, belief in one’s own abilities, trust in other people, and the ability to engage fully in relationships, leading to lower social and occupational coping (33).

Causality can also work in the opposite direction. People who are exposed to bullying may to a greater extent than other people represent some form of vulnerability in the first place. For example, it is generally accepted that physical appearance, diversity, learning disability and social status are common causes of child bullying (34). On the preventive side, education and competence in social relations or working life can protect against bullying (34).

### Health problems and life quality

The finding of more mental as well as somatic health problems among those who had been exposed to bullying is consistent with other studies in, for example, Norwegian and Australian populations (30, 35). The same applies to the finding of a lower HRQoL (30).

Least surprising, based on previous studies of child or workplace bullying, are the findings of high rates of depression (10, 11), insomnia (12, 13), anxiety (9, 14), eating disorder (15), self-harm (16), and suicidal attempts (7) among people exposed to bullying. Less well known, although previous reported, is the increased risk of psychosis (36).

The associations between bullying and somatic health problems were generally less strong than for mental health problems. The associations with somatic health were strongest for pain conditions such as chronic pain and fibromyalgia, which have been found to be associated with child or workplace bullying in several studies (19–21, 37). For some disorders such as respiratory disease and diabetes mellitus, causality can go both ways. For example, studies of children and young people have shown that those with asthma or diabetes were more likely to be exposed to bullying (22, 23). On the other hand, stress triggered by bullying can be a causal factor by interfering with the immune system (38) or triggering bronchoconstriction and asthma (39).

### Strengths and limitations

Strengths of the study include a large national probability sample of the Norwegian population, and in addition to an inquiry about bullying, questions about a wide range of mental and somatic health problems. The study was part of a larger health survey, which makes it less likely that there is a response bias based on a particular interest in bullying. However, we cannot rule out a selection bias related to participants’ willingness to take part in a health survey on a more general basis. The response rate was low, although similar to response rates usually obtained in large population surveys (40). We are unable to directly address the risk of selection bias related to bullying exposure in our sample, and this represents a limitation. Previous analyses have indicated that the sample was similar to population metrics with regards to age and sex distributions and the proportions living in urban and rural areas. However, the sample education level was somewhat higher compared to that in the general population (41, 42).

Missing data, although relatively minor (between 0 and 6.9% for the employed variables), also constitutes a study limitation, as it introduces a degree of uncertainty regarding prevalence measures of lifetime bullying in specific groups and how bullying relates to specific health problems.

The measurement of bullying consisted of a single-item approach, which is considered a valid and effective measurement of bullying (28). The measurement has been validated in large workplace populations, but lacks similar validations in general populations, which should be noted as a limitation. As opposed to, e.g., the Multiple Peer-Victimization Scale (43) used in a study on school children (8), the one-item measure does not include details of type or severity of bullying, rendering specific aspects of the bullying unclear. Also, the inability to distinguish cyberbullying from traditional bullying represents a limitation.

The ability to remember negative experiences after many years must be problematized. Consistency in reporting bullying has been shown over a 12–14-month period (44), while knowledge of what happens over many years is lacking.

Results about self-reported mental and somatic health problems have obvious limitations. They basically represent the respondent’s own experience, and there may be significant variations in judgment standards among different respondents. Nevertheless, single-item self-report measures have been shown to be reliable, as estimated by test–retest correlations (45) and correlations with clinical diagnosis (46). Discussions of causality must be considered hypothesis-generating, given the limitations of the study’s cross-sectional design.

## Conclusion and implications

The study suggests that bullying is common in the general Norwegian population, where more than a quarter of the respondents reported that they had been exposed to bullying during their lifespan. More mental and somatic health problems among those exposed to bullying may result in substantial cost for individuals, their families and society at large. Possible causal relationships between exposure to bullying and the negative health effects call for broad efforts against bullying in central arenas, such as school, work, neighborhood, and leisure activities.

Increased knowledge about the prevalence of bullying and its possible negative consequences on health and quality of life can contribute to regulating behavior and have a preventive effect on bullying. Health professionals must be aware of possible coincidences and be able to address the problem of bullying where appropriate (47). Future research should include longitudinal studies that can establish more reliable hypotheses about the connection between bullying and health in all phases of life.

## Data Availability

The raw data supporting the conclusions of this article will be made available by the authors, without undue reservation.
